# Combining heat stress and moderate hypoxia reduces cycling time to exhaustion without modifying neuromuscular fatigue characteristics

**DOI:** 10.1007/s00421-014-2883-0

**Published:** 2014-04-19

**Authors:** Olivier Girard, Sébastien Racinais

**Affiliations:** 1 Athlete Health and Performance Research Centre, Aspetar, Qatar Orthopaedic and Sports Medicine Hospital, PO Box 29222, Doha, Qatar; 2 ISSUL, Institute of Sport Sciences, Department of Physiology, Faculty of Biology and Medicine, University of Lausanne, Lausanne, Switzerland

**Keywords:** Temperature, Altitude, Peripheral fatigue, Exercise capacity

## Abstract

**Purpose:**

This study investigated the isolated and combined effects of heat [temperate (22 °C/30 % rH) vs. hot (35 °C/40 % rH)] and hypoxia [sea level (FiO_2_ 0.21) vs. moderate altitude (FiO_2_ 0.15)] on exercise capacity and neuromuscular fatigue characteristics.

**Methods:**

Eleven physically active subjects cycled to exhaustion at constant workload (66 % of the power output associated with their maximal oxygen uptake in temperate conditions) in four different environmental conditions [temperate/sea level (control), hot/sea level (hot), temperate/moderate altitude (hypoxia) and hot/moderate altitude (hot + hypoxia)]. Torque and electromyography (EMG) responses following electrical stimulation of the tibial nerve (plantar-flexion; soleus) were recorded before and 5 min after exercise.

**Results:**

Time to exhaustion was reduced (*P* < 0.05) in hot (−35 ± 15 %) or hypoxia (−36 ± 14 %) compared to control (61 ± 28 min), while hot + hypoxia (−51 ± 20 %) further compromised exercise capacity (*P* < 0.05). However, the effect of temperature or altitude on end-exercise core temperature (*P* = 0.089 and *P* = 0.070, respectively) and rating of perceived exertion (*P* > 0.05) did not reach significance. Maximal voluntary contraction torque, voluntary activation (twitch interpolation) and peak twitch torque decreased from pre- to post-exercise (−9 ± 1, −4 ± 1 and −6 ± 1 % all trials compounded, respectively; *P* < 0.05), with no effect of the temperature or altitude. M-wave amplitude and root mean square activity were reduced (*P* < 0.05) in hot compared to temperate conditions, while normalized maximal EMG activity did not change. Altitude had no effect on any measured parameters.

**Conclusion:**

Moderate hypoxia in combination with heat stress reduces cycling time to exhaustion without modifying neuromuscular fatigue characteristics. Impaired oxygen delivery or increased cardiovascular strain, increasing relative exercise intensity, may have also contributed to earlier exercise cessation.

## Introduction

Inferior performance commonly occurs during endurance-like exercises performed in hot (Ftaiti et al. 2010; Galloway and Maughan 1997; Mitchell et al. 2013; Rowland et al. 2007) or hypoxic (Amann et al. 2007; Goodall et al. 2012) compared to temperate environments at sea level. Cardiovascular factors including a lower cardiac output and a decrease in systemic blood flow impair endurance capacity in the heat (Gonzalez-Alonso et al. 2008). Acute hypoxia compromises oxygen delivery to vital organs and imposes a threat to exercise capacity by exacerbating diaphragm and abdominal muscle fatigue (Verges et al. 2010) or accentuating muscle and/or brain de-oxygenation trends (Goodall et al. 2012; Rasmussen et al. 2010). However, with major sport competitions and military efforts geographically expanding around the world some individuals are likely to exercise at moderate altitudes in the range of 2,000–3,000 m with ambient temperature sometimes exceeding 30 °C (e.g. Ifrane, Morocco; Iten, Kenya). Although exercise capacity and associated physiological, thermoregulatory and perceptual responses are generally hampered when exercise is performed with an elevated environmental temperature (Nybo 2008) or under moderate altitude conditions (Amann 2011), studies having directly compared those environmental conditions in the same individuals are scarce in the literature.

Reduction in exercise capacity in challenging environments is determined to a certain extent by the nature and/or degree of end-exercise neuromuscular fatigue experienced during the task, with specific hypoxia and heat-related effects (Amann 2011; Nybo 2008). For instance, Romer et al. (2007) reported that the level of end-exercise peripheral quadriceps fatigue in mild hypoxia was similar to that obtained in normoxia, despite time to exhaustion being reduced by more than two-third in hypoxia. However, when twelve volunteers performed a maximal incremental exercise in hot and temperate environments, early exercise cessation occurred in the hot trial along with higher core temperature and heart rate values but lower peripheral fatigue levels (Racinais and Girard 2012). To date, whether exercise capacity and the integrity of the neuromuscular system are further compromised when both environmental stressors are combined during exercise is unknown.

The intention of this study was therefore to investigate the isolated and combined effects of heat stress and moderate hypoxia on cycling time to exhaustion, associated physiological exercise responses and resulting neuromuscular adjustments. We firstly hypothesized that the combination of temperature and altitude compared to each environmental stressor alone would negatively affect exercise capacity. It has previously been shown that peripheral impairments are generally more pronounced after shorter high-intensity tasks, whereas central alterations increase with task duration (Amann 2011; Nybo 2008). As such, we further hypothesized that increasing environmental stress levels (thereby increasing exercise intensity) will accelerate the rate of development of peripheral fatigue experienced by the plantar flexors, so that end-exercise locomotor muscle fatigue will be consistent across exercise bouts. Because athletes and military personnel are regularly exposed to a wide array of environmental conditions our results could be potentially useful to design acclimation protocols that combine environmental challenges (e.g. live at altitude and exercise in combined hot and hypoxic environments) to produce greater or more rapid physiological adaptations, which in turn may boost locomotor performance.

## Methods

### Subjects

Eleven unacclimatized, physically active males (mean ± SD; age 29.0 ± 1.5 years, body weight 78.4 ± 2.0 kg, stature 179 ± 8 cm, training frequency 6.3 ± 0.7 h week^−1^) volunteered to participate in the study. Participants were instructed to avoid caffeine for 12 h and strenuous exercise for 48 h preceding each trial. This research project conformed to the standards set by the latest revision of the Declaration of Helsinki and was approved by the scientific committee of the Research and Education Centre and by the Ethics Committee of Aspetar—Qatar Orthopaedic and Sports Medicine Hospital. All subjects gave written informed consent prior to the commencement of the study, once they have received the explanation about the experimental procedures, associated risks, and potential benefits of participation.

### Experimental design

**Fig. 1:**
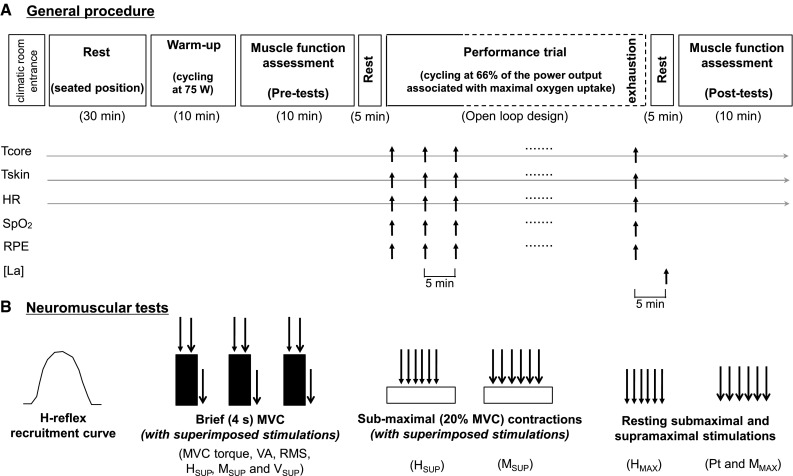
Protocol overview. General procedure (**a**) and neuromuscular assessment procedure (**b**). *Tcore* core temperature, *Tskin* skin temperature, *HR* heart rate, *SpO*_*2*_ arterial saturation percentage, *RPE* rating of perceived exertion, [*La*] blood lactate concentration, *MVC* maximal isometric voluntary contraction torque of plantar flexors. *Straight arrows* indicate the timing of motor nerve stimulations at submaximal (H-reflex, *downwards arrow*) or supra-maximal (M-wave, *downwards arrow*) intensities. Maximal H-reflex (*H*_MAX_) and M-wave (*M*_MAX_) were evoked on a relaxed muscle. The stimulation necessary to obtain *H*_MAX_ at rest was superimposed to MVC to record *H*_SUP_. When supra-maximal stimulus was superimposed to MVC, superimposed M-wave (*M*_SUP_) and V-wave (*V*_SUP_) were recorded

Each participant completed a familiarization session and four experimental trials during which they cycled to exhaustion in four different environmental conditions [temperate/sea level (control), hot/sea level (hot), temperate/moderate altitude (hypoxia) and hot/moderate altitude (hot + hypoxia)]. Temperate and hot conditions were 22 °C/30 % rH and 35 °C/40 % rH, respectively. Sea level (FiO_2_ 0.21) and moderate altitude (FiO_2_ 0.15) corresponded to a simulated altitude of ~0 and ~2,500 m, respectively. The trials were randomized, separated by at least 5–7 days, and performed at the same time of the day (±2 h) with subjects wearing shorts and t-shirts. As depicted in Fig. 1a, each experimental session was conducted as follows: (1) rest in a seated position for 30 min inside the climatic chamber, while participants were instrumented; (2) 10-min warm-up on a computer-controlled electrically braked cycle ergometer (Excalibur Sport, Lode, Netherlands) at 75 W (pedalling rate 70–80 rpm); (3) neuromuscular tests (pre-tests; ~10 min); (4) 5-min rest; (5) time trial to the limit of exhaustion at a fixed workload, equal to 66 % of the power output associated with their maximal oxygen uptake (180.5 ± 23.6 W; pedalling rate 80–90 rpm); (6) 5-min recovery including 90 s of low-intensity (50 W, 60–70 rpm) pedalling followed by 3-min rest (time for the participants to be seated on the test ergometer) and (7) neuromuscular tests (post-tests; ~10 min). Cycling exhaustive bouts began exactly 20 min after the end of the warm-up and was preceded by a 2-min low-intensity phase at the same work rate as the one used for warm-up. Constant visual and vocal feedbacks were given to the subjects to avoid variations in pedal cadence. Exercise was terminated when pedal cadence dropped below 60 rpm for >5 s (exhaustion). The participants were unaware of the experimental hypotheses and naïve to the purpose of the study. All tests (cycling and neuromuscular assessment) were performed in an environmental chamber (Tescor, Warminster, PA, USA).

### Testing procedures

#### Preliminary session

At a preliminary visit to the laboratory, subjects were first requested to perform maximal voluntary contractions (MVC) of the plantar flexors until they felt accustomed to the equipment; the coefficient of variation in three successive trials was <5 %. Afterwards, passive isometric recruitment curves (soleus M-wave and H-reflex responses to tibial nerve stimulation) were obtained. In brief, the stimulation intensity was progressively increased by 10 mA increments until there was no further increase in peak twitch torque and concomitant maximal M-wave amplitudes. This intensity was further increased by 50 % (e.g. supra-maximal) and subsequently maintained for the entire session (mean 135.0 ± 20.6 mA; range 70–200 mA). This stimulation (*M*_MAX_) allowed stable V-wave measurements (Racinais et al. 2013). The intensity needed to obtain the maximal soleus H-reflex response (*H*_MAX_ intensity) was carefully determined by re-examining the stimulus range around the *H*_MAX_ value with a precision of 1 mA (mean 55.7 ± 18.6 mA; range 35–108 mA). Thereafter, subjects performed the complete procedure of neuromuscular tests (see below). Finally, they performed a maximal incremental cycling exercise test (+25 W every minute starting from 50 W until volitional exhaustion) for the determination of maximal oxygen uptake (Quark b2, Cosmed, Rome, Italy; 46.6 ± 8.2 ml min^−1^ kg^−1^) and associated peak power output (318.2 ± 42.0 W).

#### Neuromuscular tests

The neuromuscular tests are described in Fig. 1b. The stimulation intensity needed to obtain *H*_MAX_ was re-adjusted before each recording session with a simplified procedure based on the intensity used during the preliminary session (Rupp et al. 2010). Thereafter, subjects were instructed to perform three MVCs (duration 5 s, recovery 60 s) of the plantar-flexion muscles. During each MVC, a stimulus was delivered at *H*_MAX_ intensity ~1.5 s after the beginning of the contraction (when the subject had reached a plateau) to record superimposed H-reflex (*H*_SUP_). An electrical stimulus at supra-maximal intensity delivered ~2 s after *H*_SUP_, allowed us to record the superimposed maximal M-wave (*M*_SUP_) and the V-wave (*V*_SUP_). A potentiated twitch was delivered at *M*_MAX_ intensity 5 s after the end of the MVC. Afterwards, six H-reflexes (*H*_SUP_) and six M-waves (*M*_SUP_) were evoked during a 10-s, constant (20 % of the MVC of the familiarization trial) muscle contraction. Finally, six H-reflexes (*H*_MAX_) and six M-waves (*M*_MAX_), interspaced by 15 and 10 s, respectively, were elicited from the relaxed muscle.

### Measurements

#### Exercise responses

Percent arterial oxygen saturation (SpO_2_, %) and heart rate (HR, bpm) were monitored non-invasively by pulse oximetry (IVAC Vital Care DOX Model 506 DXNT, Criticare System, Inc, USA). Rating of perceived exertion (RPE) was obtained using the 6–20 Borg scale. Core (Tcore) and skin (Tskin) temperatures were monitored via the Vitalsense system (precision 0.01 °C, Mini Mitter, Respironics, Herrsching, Germany) using an ingestible thermometer pill (swallowed 5 h before each trial) and an adhesive temperature patch on the right gastrocnemius medialis. Although a single value measurement site might not be reflective of mean skin temperature using multiple sites across the body, the skin temperature response to hot ambient condition has previously been described and we therefore recorded the temperature over the leg performing the neuromuscular test only. Finally, a capillary blood sample was taken from the fingertip and analysed for blood lactate concentration with the Lactate Pro (LT-1710, Arkray, Japan) portable analyser, exactly 5 min after exhaustion (at the commencement of neuromuscular tests).

#### Torque measurement

Isometric plantar-flexion torque of the right foot was measured using a dynamometric pedal (Captels, St Mathieu de Treviers, France). The subject’s seating position was standardized with pelvis, knee and ankle angulations of 90°, the foot securely strapped on the pedal by three straps, and a motionless head.

#### Electromyography (EMG)

EMG signals were recorded via bipolar Ag/AgCl electrodes (Ambu Blue sensor T, Ambu A/S, Ballerup, Denmark) with a diameter of 9 mm and an inter-electrode distance of 3 cm. Before electrode placement, the skin was lightly abraded and washed to remove surface layers of dead skin, hair, and oil. Subjects kept electrodes on their skin throughout the duration of the entire experiment; nevertheless, the positions of the electrodes were marked in case an electrode was lost and had to be replaced. Recording electrodes were placed on the muscle belly of the soleus and tibialis anterior muscles. A reference electrode was attached to the left wrist. The myoelectric signal was amplified (gain = 1,000×), filtered (bandwidth frequency = 30–500 Hz) and recorded (sampling frequency = 2,000 Hz) using MP35 hardware (Biopac Systems Inc., USA) and dedicated software (BSL Pro Version 3.6.7, Biopac Systems Inc, USA).

#### Stimulation procedure

The tibial nerve was stimulated by a cathode electrode with a diameter of 9 mm stuck in the popliteal cavity with constant compression supplied by a strap. The anode (5 × 10 cm, Medicompex SA, Ecublens, Switzerland) was positioned distal to the patella. Electrical stimulations (400 V, rectangular pulse of 0.2 ms) were delivered by a high-voltage stimulator (Digitimer DS7AH, Digitimer, Hertfordshire, UK).

### Data analysis

Thermoregulatory (Tcore and Tskin), physiological (HR and SpO_2_) and perceptual (RPE) responses were determined at the onset of exercise (1 min after the start), every 5 min from the fifth minute until exhaustion, and at the point of volitional exhaustion. The rate of change in these variables was estimated using linear regressions from exercise data collected at 5-min interval.

During the MVCs, torque production was recorded during a 1-s plateau prior to delivering the motor nerve stimulation. Mechanical responses to resting twitches were analysed for maximal amplitude (Pt). Voluntary activation was defined as follows: (VA, %) = [(1 − (superimposed twitch/potentiated twitch)] × 100). For voluntary contractions, the soleus or tibialis anterior root mean square (RMS) was calculated over the 1-s period of the maximal torque. Raw soleus RMS values were normalized to *M*_SUP_ (RMS/*M*_SUP_ ratio). The following parameters were determined from the electrically evoked contractions: peak-to-peak amplitude of maximal H-reflex (at rest: *H*_MAX_; during submaximal and MVC: *H*_SUP_), maximal M-wave (at rest: *M*_MAX_; during submaximal and MVC: *M*_SUP_), and V-wave (during MVC: *V*_SUP_). The *H*_MAX_/*M*_MAX_, *H*_SUP_/*M*_SUP_ (during submaximal and MVC) and *V*_SUP_/*M*_SUP_ ratios were then calculated to assess the proportion of motor units activated by the Ia afferents (Duclay and Martin 2005). For all MVCs or electrically evoked waves and associated twitches, the values of three or six trials, respectively, were averaged for subsequent analysis.

### Statistical analysis

Data are presented as mean ± SD. Normal distribution of the data was tested using the Kolmogorov–Smirnov test. Sphericity (homogeneity of covariance) was verified by the Mauchly’s test. When the assumption of sphericity was not met, the significance of *F*-ratios was adjusted according to the Greenhouse–Geisser procedure. A three-way analysis of variance (time × temperature × altitude) with repeated measures was used to compare exercise data. Neuromuscular variables were compared by a three-way analysis of variance (exercise × temperature × altitude) for repeated measures. Statistical analyses were undertaken using the SPSS statistical package (version 15.0, SPSS, Chicago, IL, USA). Statistical significance was accepted at *P* < 0.05.

## Results

### Time to exhaustion

**Fig. 2:**
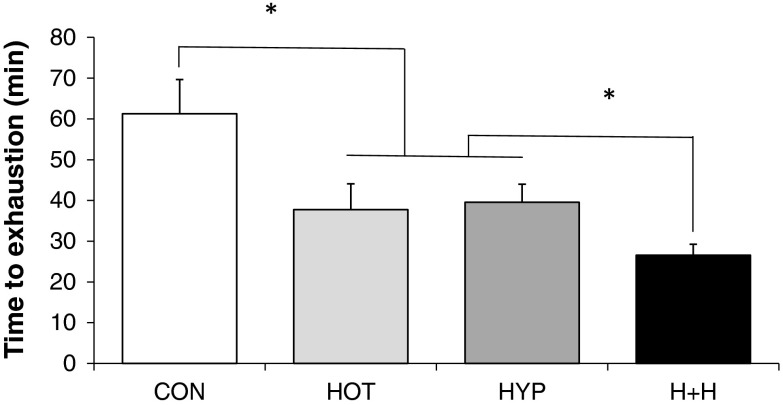
Time to exhaustion in four different environmental conditions. *CON* temperate and sea level, *HOT* hot and sea level, *HYP* temperate and moderate altitude, *H* *+* *H* hot and moderate altitude. *P < 0.05. Temperature and altitude had a main effect (*P* < 0.05) on time to exhaustion

Temperature (*P* < 0.05) and altitude (*P* < 0.05) both reduced time to exhaustion (Fig. 2), while there was also a significant interaction effect (*P* < 0.05) between these two factors. Hot + hypoxia was shorter than hot or hypoxia alone (*P* < 0.05). There was an antagonistic effect of either a hot (−22 ± 4 % from control to hot vs. −11 ± 3 % from hypoxia to hot + hypoxia; *P* < 0.05) or an altitude (−24 ± 6 % from control to hypoxia vs. −13 ± 5 % from hot to hot + hypoxia; *P* < 0.05) exposure when the other stressor was already present.

### Exercise responses

#### Exercise onset

After the first minute of exercise, HR (*P* < 0.05) and Tskin (*P* < 0.05) were higher in hot conditions, whereas altitude exposure reduced SpO_2_ (*P* < 0.05) and increased HR (*P* < 0.05). Neither temperature nor altitude had a significant influence on Tcore and RPE at exercise commencement.

#### Rate of change

During exercise, hot conditions increased (*P* < 0.05) the rate of rise in Tcore, HR and RPE (Table 1). Altitude exposure induced a faster rate of increase (*P* < 0.05) in HR and RPE and of decrease (*P* < 0.05) in SpO_2_. There was no significant interaction effect (*P* > 0.05) between temperature and altitude on any parameter reflecting responses to exercise.

**Table 1:** Effects of temperature [temperate (22 °C/30 % rH) vs. warm (35 °C/40 % rH)] and altitude [sea level (FiO_2_ 0.21/simulated altitude ~0 m) vs. moderate altitude (FiO_2_ 0.15/simulated altitude ~2,500 m)] on the rate of change in exercise responses

	Temperature		Altitude	
	Temperate	Warm	Sea level	Moderate altitude
Tcore (°C/min)	0.030 ± 0.002	0.050 ± 0.005*	0.037 ± 0.004	0.043 ± 0.004
Tskin (°C/min)	0.042 ± 0.008	0.035 ± 0.005	0.034 ± 0.005	0.043 ± 0.006
HR (beats/min)	1.468 ± 0.196	2.382 ± 0.242*	1.423 ± 0.202	2.427 ± 0.239^#^
SpO_2_ (%/min)	−0.085 ± 0.029	−0.117 ± 0.068	−0.013 ± 0.010	−0.189 ± 0.075^#^
RPE (point/min)	0.269 ± 0.037	0.426 ± 0.048*	0.249 ± 0.037	0.445 ± 0.045^#^

Values are expressed as mean ± SEM

*Tcore* core temperature, *Tskin* skin temperature, *HR* heart rate, *SpO*_*2*_ arterial oxygen saturation, *RPE* rating of perceived exertion

* *P* < 0.05; significantly higher in warm than in temperate conditions (temperature main effect)

^#^ *P* < 0.05; significantly higher in moderate altitude than in sea level conditions (altitude main effect)

#### Exhaustion

**Fig. 3:**
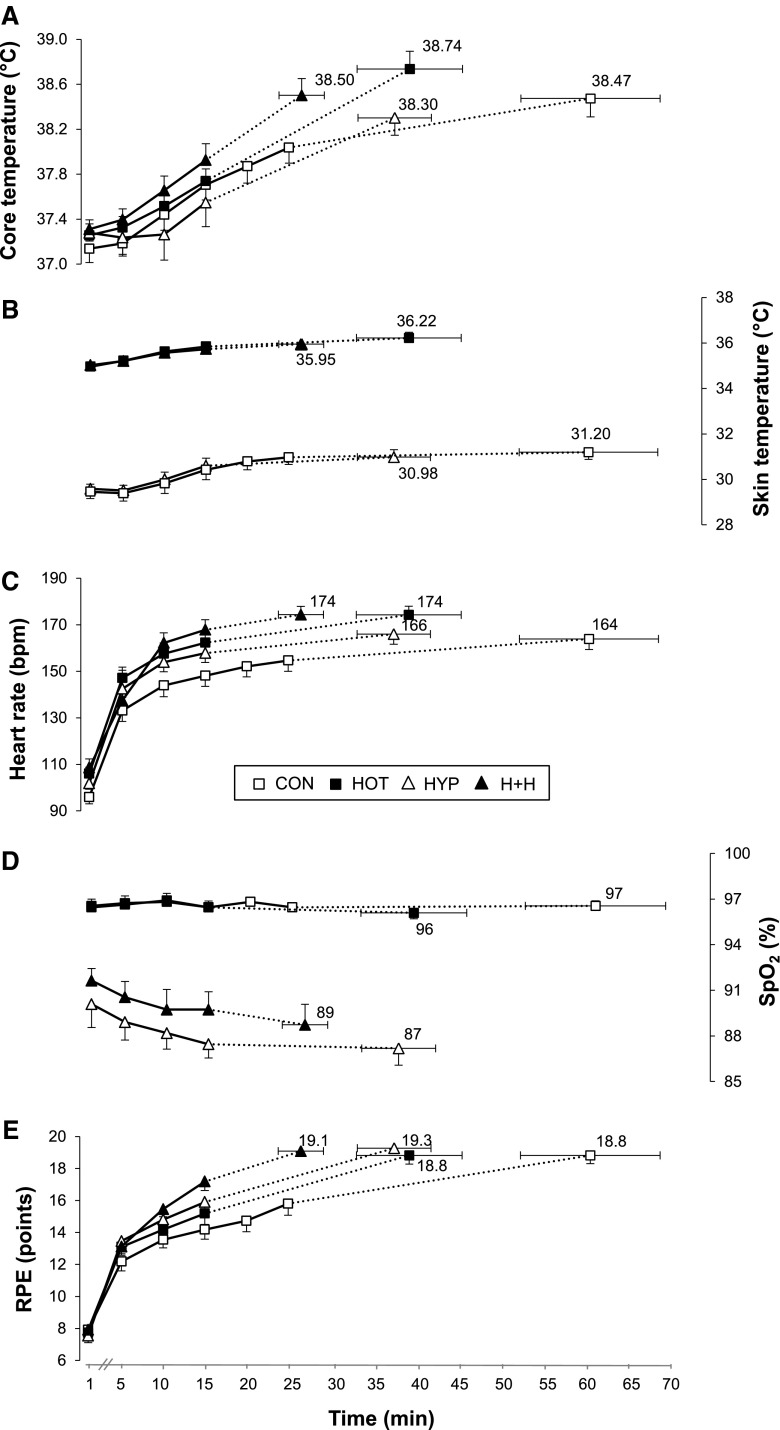
Exercise responses in four different environmental conditions. *CON* temperate and sea level, *HOT* hot and sea level, *HYP* temperate and moderate altitude, *H* *+* *H* hot and moderate altitude. See text for details. At the onset of exercise, HR and Tskin were higher (*P* < 0.05) in warm conditions, whereas altitude exposure reduced SpO_2_ and increased HR (both *P* < 0.05). Warm environments increased Tskin and HR at exhaustion (*P* < 0.05), whereas SpO_2_ (*P* < 0.05) was lowered with altitude exposure. The rate of rise in Tcore, HR and RPE was speeded under warm conditions (*P* < 0.05), whereas the rate of HR and RPE increase and SpO_2_ decrease was faster with altitude exposure (both *P* < 0.05). There was no interaction effect between temperature and altitude on either parameter

Hot conditions significantly (*P* < 0.05) increased HR (+9 ± 5 bpm) and Tskin (+5 ± 1 °C) at exhaustion independently of the simulated altitude, whereas altitude exposure reduced SpO_2_ independently of the environmental temperature (96 ± 1 vs. 88 ± 4 % in sea level and moderate altitude conditions, *P* < 0.05) (Fig. 3). There was no main effect of temperature and altitude on Tcore (*P* = 0.089 and *P* = 0.070, respectively) or RPE (*P* > 0.49).

When measured +5 min after exhaustion, significantly higher (*P* < 0.05) blood lactate concentration values were reached in environments with moderate altitude (5.6 ± 1.0, 7.9 ± 0.5, 8.7 ± 2.6 and 10.0 ± 0.6 mmol L^−1^ in control, hot, hypoxia and hot + hypoxia, respectively), whereas no temperature main effect nor any significant interaction was observed.

### Neuromuscular data

**Fig. 4:**
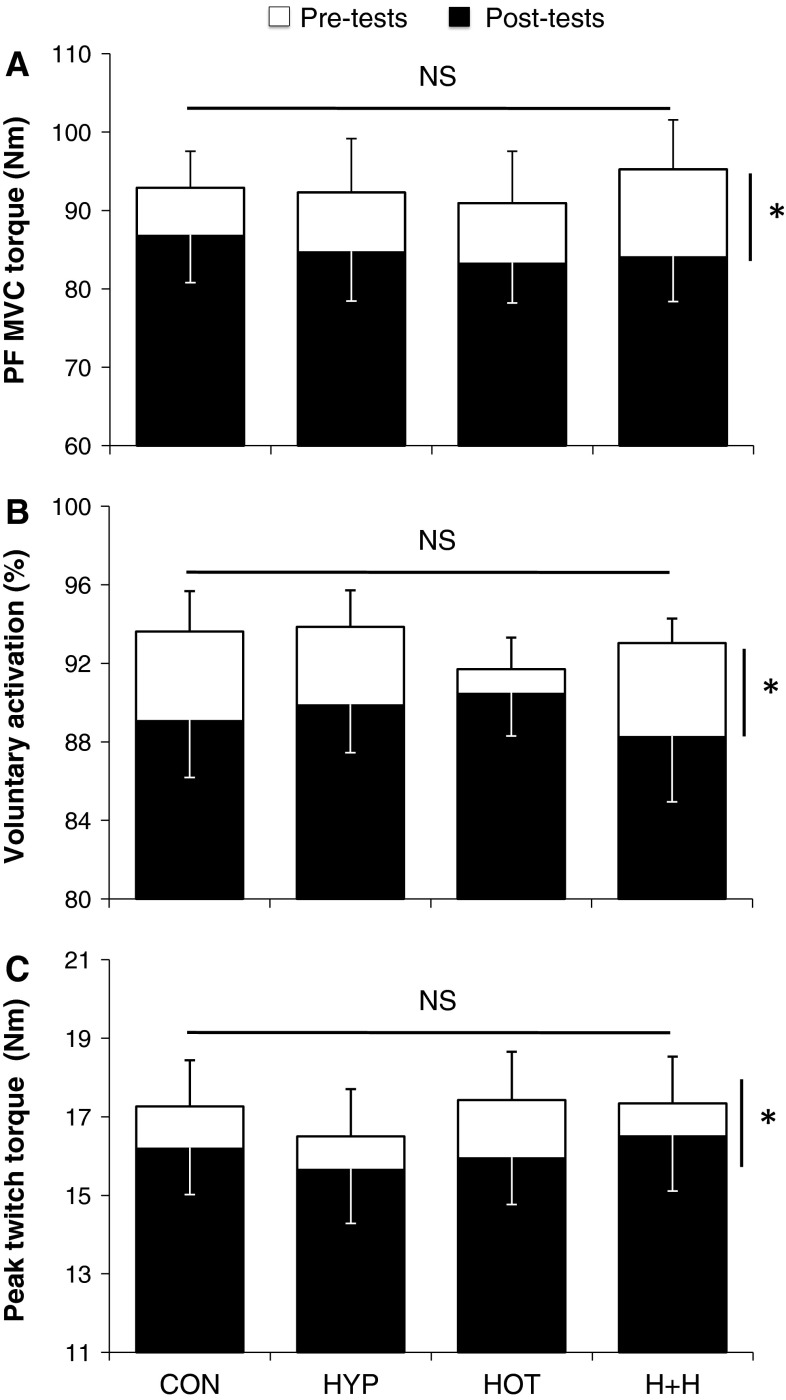
Maximal isometric voluntary contraction torque of plantar flexors (PF MVC torque, **a**), voluntary activation (twitch interpolation technique, **b**) and peak twitch torque (Pt, **c**) before (pre-tests) and after (post-tests) cycling to exhaustion in various conditions. *CON* temperate and sea level, *HOT* hot and sea level, *HYP* temperate and moderate altitude, *H* *+* *H* hot and moderate altitude.*P < 0.05, significantly different from pre-tests. There was neither a main effect of temperature or altitude nor any interaction between temperature and altitude on either parameter

Significant reductions occurred from pre- to post-exercise for MVC torque, VA and Pt (−8.6 ± 1.5, −3.9 ± 1.5 and −6.1 ± 0.8 %, respectively; all trials compounded; *P* < 0.05), independently of the environmental conditions (*P* < 0.05; Fig. 4). Table 2 presents EMG-related variables determined during the neuromuscular assessment performed before and +5 min after the fatigue protocol in various conditions. Raw *H*_MAX_ (*P* < 0.05) and *H*_MAX_/*M*_MAX_ ratio values were significantly (*P* < 0.05) reduced from pre- to post-exercise, with no temperature or altitude main effects. Compared to temperate environments, the amplitude of both M-waves (at rest, during submaximal contraction and MVC) and V-waves, but not H-reflexes, together with raw soleus RMS activity was significantly reduced (*P* < 0.05) in hot conditions (Table 2). There was neither a significant main effect of altitude nor a significant interaction between exercise, altitude and/or temperature for any normalized EMG-related variables.

**Table 2:** EMG-related variables assessed before (pre-tests) and after (post-tests) cycling to exhaustion in various conditions

	Pre-tests				Post-tests				ANOVA
	Control	Hot	Hypoxia	Hot + hypoxia	Control	Hot	Hypoxia	Hot + hypoxia	
Rest									
*M*_MAX_ (mV)	8.75 ± 2.93	5.63 ± 1.56	7.20 ± 2.90	5.38 ± 2.05	8.25 ± 2.59	5.72 ± 1.47	7.07 ± 2.52	5.14 ± 2.04	*T*
*H*_MAX_ (mV)	3.31 ± 2.15	3.37 ± 1.64	3.07 ± 2.06	2.23 ± 1.57	2.78 ± 2.14	1.89 ± 1.13	2.57 ± 1.76	1.96 ± 1.41	*E*
*H*_MAX_/*M*_MAX_ (%)	38.3 ± 18.8	59.5 ± 24.2	41.2 ± 17.8	43.2 ± 31.3	32.4 ± 19.8	33.0 ± 17.9	36.9 ± 19.5	40.7 ± 37.6	*E*
20 % MVC									
*M*_SUP_ (mV)	8.88 ± 3.63	6.06 ± 1.74	7.53 ± 2.58	5.97 ± 2.40	8.59 ± 2.80	6.19 ± 2.12	7.26 ± 2.57	5.89 ± 2.04	*T*
*H*_SUP_ (mV)	3.51 ± 2.24	2.76 ± 1.40	3.15 ± 1.91	2.44 ± 1.32	3.36 ± 2.31	2.72 ± 1.01	2.68 ± 1.89	2.40 ± 1.63	
*H*_SUP_/*M*_SUP_ (%)	41.4 ± 18.5	43.6 ± 14.2	41.3 ± 16.0	43.8 ± 24.3	39.3 ± 22.8	45.0 ± 12.4	38.0 ± 23.1	39.5 ± 27.0	
100 % MVC									
*M*_SUP_ (mV)	9.73 ± 2.73	6.36 ± 1.64	7.76 ± 2.57	5.93 ± 1.95	8.21 ± 2.57	7.21 ± 2.41	7.97 ± 2.60	6.28 ± 2.17	*T*
*H*_SUP_ (mV)	3.99 ± 2.00	2.79 ± 1.40	3.10 ± 1.43	2.92 ± 1.52	4.34 ± 1.82	3.46 ± 1.03	3.30 ± 1.54	3.06 ± 1.26	
*H*_SUP_/*M*_SUP_ (%)	39.4 ± 11.1	42.1 ± 18.1	42.9 ± 20.6	50.7 ± 23.5	51.7 ± 13.6	49.9 ± 15.6	42.7 ± 19.0	50.9 ± 21.8	
*V*_SUP_ (mV)	2.35 ± 1.79	1.61 ± 0.81	1.98 ± 0.92	1.72 ± 0.70	2.11 ± 1.27	1.58 ± 0.89	1.86 ± 1.02	1.53 ± 1.13	*T*
*V*_SUP_/*M*_SUP_ (%)	22.2 ± 13.0	24.5 ± 11.1	25.7 ± 8.2	31.1 ± 14.3	24.9 ± 11.5	23.9 ± 17.8	23.8 ± 11.4	23.8 ± 14.4	
RMS TA (mV)	0.047 ± 0.018	0.043 ± 0.011	0.046 ± 0.013	0.043 ± 0.007	0.042 ± 0.016	0.039 ± 0.013	0.041 ± 0.012	0.037 ± 0.017	*E*
RMS SOL (mV)	0.287 ± 0.138	0.195 ± 0.070	0.254 ± 0.076	0.201 ± 0.080	0.261 ± 0.138	0.196 ± 0.077	0.233 ± 0.081	0.186 ± 0.070	*E*, *T*
RMS/*M*_SUP_ (au)	0.029 ± 0.009	0.032 ± 0.010	0.035 ± 0.011	0.034 ± 0.007	0.033 ± 0.015	0.028 ± 0.008	0.030 ± 0.013	0.031 ± 0.013	

Values are expressed as mean ± SD. Subjects cycled to exhaustion in four different environmental conditions [temperate/sea level (control), warm/sea level (hot), temperate/moderate altitude (hypoxia) and warm/moderate altitude (hot + hypoxia)] in which temperature [temperate (22 °C/30 % rH) vs. warm (35 °C/40 % rH)] and altitude levels [sea level (FiO_2_ 0.21/simulated altitude ~0 m) vs. moderate altitude (FiO_2_ 0.15/simulated altitude ~2,500 m)] were set

There was neither a main effect of altitude nor any interaction between temperature and altitude on either parameter

*M*_*MAX*_ maximal resting M-wave, *H*_*MAX*_ maximal resting H-reflex, *M*_*SUP*_ maximal superimposed M-wave, *H*_*SUP*_ maximal superimposed H-reflex, *V*_*SUP*_ maximal superimposed V-wave, *RMS TA* tibialis anterior root mean square, *RMS SOL* soleus root mean square

*E*, *P* < 0.05; denote a significant main effect of exercise

*T*, *P* < 0.05; denote a significant main effect of temperature

## Discussion

We examined the isolated and combined effects of environmental temperature and altitude on exercise capacity, physiological responses to exercise and neuromuscular fatigue characteristics. The most important findings were that (1) time to exhaustion during a constant-load cycling exercise with either temperature or altitude challenge is reduced by about one-third compared to a control, and by more than half when combining these two stressors, (2) the separate temperature- and altitude-induced changes in physiological, thermoregulatory and perceptual responses to exercise were independent of each other (i.e. no interaction effect) and (3) the decline in force output of the plantar flexors and the magnitude of accompanying neural (muscle activation) and muscular (peak twitch torque) adjustments were similar among all trials.

### Exercise capacity

It is already well documented that the exercise capacity for endurance-type efforts deteriorates in hot (Ftaiti et al. 2010; Galloway and Maughan 1997; Mitchell et al. 2013; Rowland et al. 2007) and hypoxic (Amann et al. 2007; Goodall et al. 2012) conditions. A novel finding, however, was that combining those stressors further compromised locomotor performance since exercise capacity was reduced by 50 % compared with similar intensity exercise in hot or hypoxic environment alone. These data show for the first time that combining heat stress and moderate hypoxia reduces cycling time to exhaustion with no additional effect on neuromuscular fatigue characteristics. Other factors (not evaluated in this study) such as increased de-oxygenation trends (Goodall et al. 2012; Rasmussen et al. 2010) or cardiovascular strain due to impairments in stroke volume and/or cardiac output (Gonzalez-Alonso et al. 2008) may have played a pivotal role in earlier exercise cessation in challenging environmental conditions.

### Exercise responses

Our results confirm that exercising in hot or hypoxic conditions has an impact on the rate of changes in physiological, thermoregulatory and perceptual responses (Amann et al. 2007; Galloway and Maughan 1997; Périard et al. 2011; Racinais and Girard 2012). Increases in HR and RPE were faster in both environments compared to control, while hot environment alone speeded the exercise-induced rise in Tcore and hypoxic exposure only accelerated the SpO_2_ decrease. While the absence of statistical difference in Tcore might be surprising, it has to be acknowledged that the current exercise model leads to shorter time to exhaustion in hot trials (i.e. the rate of increase in Tcore was higher in hot than temperate trials). We also report that the level of blood lactate concentration, as measured 5-min post-exhaustion, was elevated with altitude exposure despite nearly identical levels of muscle fatigue (see below). In line with previous time trial findings (Amann et al. 2006), these results indicate a greater anaerobic energy release under hypoxic conditions (McLellan et al. 1990).

It has recently been reported that combining environmental stressors accentuated the physiological stress (HR and RPE values) during a moderate-intensity (60 min at 40 % of maximal oxygen uptake on a treadmill) in different hot (environmental temperature = 23 vs. 35 °C) and hypoxic (FiO_2_ = 20.9 vs. 16.5 %) conditions (Buono et al. 2012). The current data showed that if the constant-load exercise is performed until exhaustion, Tcore, HR and SpO_2_ values recorded at exhaustion with heat or altitude alone did not differ from a combination of both stressors.

### Strength capacity and neural factors

In this study, strength losses in PF were relatively modest (~9 %) as compared to the losses induced by running exercises (~10–40 %; Girard et al. 2012; Millet et al. 2011; Perrey et al. 2010; Racinais et al. 2007) but were in accordance with the losses previously observed after a maximal cycling exercise (~8 %; Racinais and Girard 2012). Maximal EMG activity and twitch interpolation recordings obtained during MVC were used to assess the completeness of plantar flexors activation capacity. Our results displayed a ~7 % reduction in maximal, raw soleus RMS activity, whereas reductions (even though of similar extent) for the normalized EMG signal (RMS/*M*_SUP_ ratio) did not reach significance. In addition, VA decreased consistently from ~93 to 89 % across trials. In line with previous studies showing that plantar flexor muscles are subjected to decrements in VA in response to sustained, complex functional movements (e.g. running: Millet et al. 2011; Racinais et al. 2007; cycling: Racinais and Girard 2012; tennis match play: Girard et al. 2011), our results confirm that the neural input reaching the muscle—at least measured during MVCs—decreases with fatigue. However, surface EMG and twitch interpolation techniques do not quantify the descending drive to the lower motoneurons nor do they take into account the source of this drive (De Haan et al. 2009). Thus, other neurostimulation techniques were implemented to shed more light on the potential sites and mechanisms underlying neural adjustments to exhaustive cycling (Gruet et al. 2013).

Previous studies have reported no alteration in voluntary activation during the first seconds of an MVC (Nybo and Nielsen 2001), suggesting that participants could override central fatigue during brief MVCs. However, it is difficult to discriminate the relative role of the temperature and the exercise in these previous data (Racinais and Oksa 2010) as passive hyperthermia has been shown to alter even brief MVCs (Morrison et al. 2004; Thomas et al. 2006; Racinais et al. 2008). Notwithstanding, the muscle group investigated in the current study (i.e. PF) has been shown to be altered due to both exercise and hot ambient conditions, even during brief MVCs performed 5 min after the end of an exhaustive cycling exercise (Racinais and Girard 2012).

### Reflex responses

In our study, the reduction in resting H-reflexes amplitude from pre- to post-exercise, occurring independently of temperature or altitude, would suggest that the balance between excitation and inhibition affecting the α-motor neuron pool was altered with fatigue. Because the nature of spinal reflex modulations during passive and active conditions may differ (Racinais et al. 2013), H-reflex and V-wave responses were assessed during voluntary efforts/background contractions. In addition to reflecting both reflex excitability and presynaptic inhibition of Ia afferents (spinal processes), V-wave response is also indicative of the level of neural drive in descending corticospinal pathways (supraspinal mechanisms) (Del Balso and Cafarelli 2007). Few existing studies have shown concomitant decrements in H-reflex and V-wave responses during MVC in response to various whole-body exercises such as a 90-min run (Racinais et al. 2007) and a 3-h tennis match play (Girard et al. 2011). There are also reports of decreased H-reflex and V-wave amplitudes in response to passive hyperthermia (Racinais et al. 2008), while the impact of hypoxia on those reflex responses has not been documented yet. In this study, however, reflex responses as measured during voluntary efforts remained unchanged, indicating that neither fatigue nor heat and hypoxia had a significant impact on spinal loop modulation mechanisms.

### Peripheral factors

The present findings confirm that maximal soleus M-wave amplitudes are reduced in hot compared to temperate conditions (Racinais et al. 2008; Racinais and Girard 2012), whereas healthy subjects exposed to acute hypoxia rarely experience muscle excitability (ionic disturbances) perturbations (Perrey and Rupp 2009). This may in turn explain the reduced soleus RMS (VA values were not sensitive to heat exposure) values in hot conditions, as changes in neuromuscular transmission and/or muscle membrane propagation characteristics are likely to affect the raw EMG signal. Noteworthy, no change in soleus M-wave properties—as measured both at rest and during voluntary contractions—but a decrement in the maximal amplitude of evoked twitch torque occurred from pre- to post-exercise, which places the site of peripheral perturbations distant to the muscle membrane.

Although our experimental techniques did not allow investigating further the likely cause of this exercise-induced impeded calcium handling (i.e. excitation–contraction uncoupling; for a review see Allen et al. 2008), a remarkable finding was that the degree of environmental stress modifies the rate of peripheral fatigue development (i.e. identical magnitude of peripheral fatigue incurred at exhaustion across all four trials). When nine male subjects cycled to exhaustion at a fixed work rate (90 % of *V*O_2max_) in normoxia and severe hypoxia (FiO_2_ = 0.21 and 0.13, respectively), Romer et al. (2007) also reported a very similar (−34 vs. −39 %) end-exercise reduction in quadriceps twitch force, while endurance capacity was reduced by more than two-third with altitude exposure. This equally attenuated twitch force occurring, in this study, independent of the temperature or altitude and/or the marked differences in exercise capacity is in line with the hypothesis claiming that peripheral fatigue is a carefully regulated variable—i.e. exercise is voluntarily terminated once a specific level of peripheral fatigue (sensory tolerance limit) has been reached during whole-body cycling (Amann 2011).

It has also recently been postulated that subjects stop exercising (exhaustion) in the face of extreme hot (core temperature >39 °C; Racinais and Girard 2012) and hypoxic (SpO_2_ <75 %; Amann et al. 2006, 2007) conditions before substantial levels of peripheral fatigue are incurred. Under these circumstances, substantial levels of muscle/cerebral de-oxygenation and hypoexcitability of the cortical circuits may outweigh the limiting effects of peripheral fatigue and associated inhibitory feedback (Amann 2011; Nybo 2008). However, none of these direct sources of inhibition of central motor drive have been explicitly examined in this study, therefore preventing us to draw definitive conclusions. Although similar to what many athletes and military personnel may experience on the field, however, the heat stress (core temperature <~38.8 °C) and degree of hypoxia (SpO_2_ >~87 %) were relatively modest in the conditions of the present study. It is therefore unlikely that these ‘direct’ neural influences may have played a significant role in curtailing peripheral fatigue development when exercising in hot and/or hypoxic conditions.

### Limitations and perspectives

A few limitations of our study must be noted. Firstly, because significant recovery occurs in skeletal muscle function within the first 2 min after intense exercise, it cannot be ruled out that the 5-min period separating exhaustion to post-exercise neuromuscular tests may have caused some recovery and hence underestimated neuromuscular function alterations (Froyd et al. 2013). Of note, however, not only the extent, but also the nature of the neuromuscular fatigue induced by the constant-load exercise was similar at the termination of exercise. Moreover, the possible priming effect of ~40 min of intervention, potentially affecting the neuromuscular system before pre-tests of muscle function and cycling time trial, should not be overlooked.

Secondly, it is unclear whether fatigue mechanisms identified during maximal isometric contractions of a single muscle group [i.e. plantar flexors with EMG analysis of one ankle extensors (soleus) and one ankle flexor (tibialis anterior) muscle] and during a complex functional movement (i.e. cycling to the limit of tolerance at a submaximal constant work rate) are comparable.

Thirdly, we chose to study PF because fatigue-induced neuromuscular adjustments have previously been documented in this particular muscle group after intense cycling (Racinais and Girard 2012), hence allowing a careful evaluation of reflex responses. Nevertheless, whether similar or more pronounced alterations in neuromuscular fatigue characteristics occur in other muscle groups largely involved in cycling (e.g. knee extensors) still needs to be researched. Furthermore, EMG recordings during cycling would be needed to confirm that the relative level of central motor output required for a fixed cycling work rate was exaggerated in the environment where heat and hypoxia were combined. This evaluation needs to be extended to other mono-articular (e.g. vastus lateralis, vastus medialis and gluteus maximus) and several bi-articular muscles (e.g. rectus femoris, semimembranosus, gastrocnemius lateralis and medialis) to carefully evaluate the pattern of muscle activation.

Lastly, the results of the present study have been obtained with a moderately trained population performing laboratory-based tests and therefore should not be generalized to highly trained athletes. Future studies adopting more face-valid tests of performance are warranted to reflect what happens on the field. Finally, the potential role of respiratory muscle fatigue in exercise capacity limitation under hypoxic conditions cannot be ruled out and needs to be investigated in future studies, since hypoxia is known to exacerbate both diaphragm and abdominal muscle fatigability (Verges et al. 2010).

## Conclusion

The present study evaluated, for the first time, the isolated and combined effects of environmental temperature and altitude on cycling time to exhaustion and neuromuscular fatigue characteristics. Our findings showed that, compared to a control, exercise capacity is reduced by about one-third when cycling to exhaustion at an absolute work rate with temperature or altitude challenge alone and by more than half when combining these two stressors. Our results also display that both the extent and the characteristics of muscle fatigue were similar at exhaustion in different environmental conditions despite different time to exhaustion. Future studies should investigate if environmental conditions affect the rate of muscle fatigue development.

## Abbreviations

EMG: Electromyography
FiO_2_: Inspired fraction of oxygen
*H*_MAX_: Maximal resting H-reflex
*H*_SUP_: Maximal superimposed H-reflex
HR: Heart rate
[La]: Blood lactate concentration
*M*_MAX_: Maximal resting M-wave
*M*_SUP_: Maximal superimposed M-wave
MVC: Maximal voluntary contraction
PF: Plantar flexors
RMS: Root mean square
RPE: Rating of perceived exertion
SpO_2_: Percent arterial oxygen saturation
Tcore: Core temperature
Tskin: Skin temperature
VA: Voluntary activation
*V*_SUP_: Maximal superimposed V-wave
